# A Simulation Study to Assess the Effect of the Number of Response Categories on the Power of Ordinal Logistic Regression for Differential Item Functioning Analysis in Rating Scales

**DOI:** 10.1155/2016/5080826

**Published:** 2016-06-15

**Authors:** Elahe Allahyari, Peyman Jafari, Zahra Bagheri

**Affiliations:** Department of Biostatistics, Faculty of Medicine, Shiraz University of Medical Sciences, Shiraz, Iran

## Abstract

*Objective.* The present study uses simulated data to find what the optimal number of response categories is to achieve adequate power in ordinal logistic regression (OLR) model for differential item functioning (DIF) analysis in psychometric research.* Methods.* A hypothetical ten-item quality of life scale with three, four, and five response categories was simulated. The power and type I error rates of OLR model for detecting uniform DIF were investigated under different combinations of ability distribution (*θ*), sample size, sample size ratio, and the magnitude of uniform DIF across reference and focal groups.* Results.* When *θ* was distributed identically in the reference and focal groups, increasing the number of response categories from 3 to 5 resulted in an increase of approximately 8% in power of OLR model for detecting uniform DIF. The power of OLR was less than 0.36 when ability distribution in the reference and focal groups was highly skewed to the left and right, respectively.* Conclusions.* The clearest conclusion from this research is that the minimum number of response categories for DIF analysis using OLR is five. However, the impact of the number of response categories in detecting DIF was lower than might be expected.

## 1. Introduction

In studies related to quality of life, measurement equivalence is an essential assumption for meaningful comparison of health-related quality of life scores across different populations. Violation of this statistical property at the item level, also known as differential item functioning (DIF), is an important part of the process of validating health-related quality of life (HRQoL) instruments [1, 2]. DIF analysis originated in educational testing is now increasingly being used in psychometric studies to assess whether the probability of responding to a specific item within a HRQoL scale differs between the compared groups after controlling the construct being measured. There are different types of DIF detection methods for Likert-type items including multiple-group categorical confirmatory factor analysis (MGCFA), item response theory (IRT), and ordinal logistic regression model (OLR) [3–8]. These methods use different assumptions and procedures to test measurement equivalence; however, they share conceptual similarities such as having ability to examine both uniform and nonuniform DIF. In this perspective, some researchers have tried to compare various DIF detection methods by focusing on real data. They found that applying various methods for examining DIF may lead to different results [9]. Beyond that, researchers designed simulation studies to get more insights into similarities and differences of DIF detection methods. Despite the existence of these stimulation studies, further attention is required to clarify some statistical properties of DIF detection methods under different conditions.

In the present study, we have just focused on OLR model as a well-known method in DIF analysis. Unlike IRT model, OLR is able to control additional covariates, both categorical and continuous, which may confound the results of DIF analysis. Moreover, it does not assume normality of the group ability and provides a number of criteria to quantify the magnitude of DIF which may not be practically important [7, 10].

Previous simulation studies have shown how various factors including sample size, sample size ratio, scale length, magnitude of DIF, and the ability distribution can affect the results of DIF analysis by OLR model [5, 10, 11]. Although the quality of an instrument changes according to the number of response options [12, 13], the same number of response categories was used in all of these simulation studies. A number of simulation studies have attempted to answer the question of what the optimal number of response options is for psychological instruments. In these researches, authors found that the optimum number of response options is between four and seven, and reliability and validity decrease with fewer than four categories [14, 15]. In addition, when the number of items is small or if the items are low discriminating, using more response categories can increase the precision of instruments. Moreover, in addition to the psychometric properties, the number of response categories may influence the level of response bias [16]. Acquiescence and extreme response styles are two types of response bias that are highly dependent on the number of response categories in a measure. The first one is defined as the tendency to agree to propositions in general, while the second one is described as the tendency for people to consistently use the extreme ends of response scales [16].

Although these studies have investigated the effect of the number of response categories on psychometric properties of psychological instruments, to our best of knowledge, this issue has not been previously evaluated on the statistical properties of OLR for detecting DIF. To fill this gap, the present study uses simulated data to find what the optimal number of response categories is to achieve an acceptable level of power and type I error rates in OLR model for DIF analysis. Hence, this study aims to investigate if the effect of the number of response categories on the power of OLR for detecting DIF can be influenced by the skewed ability distributions, sample size, sample size ratio, and the magnitude of DIF across reference and focal groups.

## 2. Methods

### 2.1. Ordinal Logistic Regression Model for Detecting DIF

Testing for the presence of uniform and nonuniform DIF under ordinal logistic regression (OLR) is based on comparing three different models as follows:

 Model 1: Logit [*P*(*Y* ≤ *K*)] = *α*_*k*_ + *β*_1_ *θ*;
 Model 2: Logit [*P*(*Y* ≤ *K*)] = *α*_*k*_ + *β*_1_ *θ* + *β*_2_ *g*;
 Model 3: Logit [*P*(*Y* ≤ *K*)] = *α*_*k*_ + *β*_1_ *θ* + *β*_2_ *g* + *β*_3_ *gθ*.

In this model, the term *θ* is the ability or observed trait level of an individual usually denoted by total test score, and *g* is the grouping variable with two levels including reference and focal groups. According to the above models, uniform and nonuniform DIF could be detected by comparing the log likelihood values for Models 1 and 2 and Models 2 and 3, respectively. For both uniform and nonuniform DIF twice the difference in log likelihoods is compared to a chi-square distribution with one degree of freedom.

For items with nonuniform DIF, the direction of DIF differs along *θ*, and consequently the effect of nonuniform DIF can be cancelled out at the scale level [17] which is the main reason for focusing on uniform DIF in this study.

### 2.2. Data Generation

In this study, the graded response model (GRM) was used to generate response data for a measure with 10 items. The mathematical form of the GRM is

(1) $$\begin{array}{c} P_{ij}\left(\;\theta \;\right)=\frac{e^{a_{i}\left(\theta -b_{ij}\right)}}{1+e^{a_{i}\left(\theta -b_{ij}\right)}}, \end{array}$$

where *P*_*ij*_(*θ*) is the probability of scoring in or above category *j* of item *i*, *a*_*i*_ is the item discrimination parameter, *b*_*ij*_ is the threshold for category *j* of item *i*, and *θ* represents the latent trait. In this study, *b*_*ij*_ parameters were simulated from the standard normal distribution, and *a*_*i*_ parameters were sampled from the uniform distribution over the interval (0.5,1.5). An item *i* with *j* categories (*j* = 1 to *J*) will be characterized by a vector of threshold parameters *b*_*ij*_ as follows:

(2) $$\begin{array}{c} b_{ij}=\left[\begin{array}{c} b_{i1}\\ b_{i2}\\ \vdots \\ b_{iJ} \end{array}\right]. \end{array}$$

*a*_*i*_ and *b*_*ij*_ parameters were fixed (for the entire simulation) within a particular simulated dataset.

In this simulation study, five factors were varied: response categories, sample size, sample size ratio, magnitude of uniform DIF, and ability distribution. The number of response categories of the items ranged from 3 to 5 (*J* = 3,4, 5). Two sample sizes (*N* = 600, *N* = 1000) and three levels of sample size ratio (*R* = 1,2, 3) were investigated. Sample size ratio between the reference and focal groups was set to 1 : 1 for the equal sample size conditions and 2 : 1 and 3 : 1 for the unequal sample size conditions. More specifically, we created conditions with *n*_*R*_/*n*_*F*_ = 300/300, 400/200, and 450/150 for the medium sample size (*N* = 600) and *n*_*R*_/*n*_*F*_ = 500/500, 667/333, 750/250 for the large sample size (*N* = 1000). Moderate and severe uniform DIF were also simulated by adding 0.5 and 1 to *b*_*ij*_ parameters in the focal group, respectively. In this study, the length of the scale was held constant at 10 and just one item with uniform DIF was simulated.

### 2.3. Simulated Distributions of the Latent Trait

The GRM, used in the present study to generate item responses, assumes the normality assumption for the latent trait (*θ*). To assess whether the impact of the number of response categories on the power of OLR can be influenced by the ability distribution, we simulated nine different *θ* distribution conditions (Table 1). In the first condition, the *θ* distribution for the reference and focal groups was a standard normal. For the other eight conditions, beta distribution—Beta (*α*, *β*)—was used to generate moderately and highly skewed ability distributions. The beta distribution is a member of continuous probability distributions defined on the interval (0,1) with two positive parameters, including *α* and *β*.

**Figure 1:**
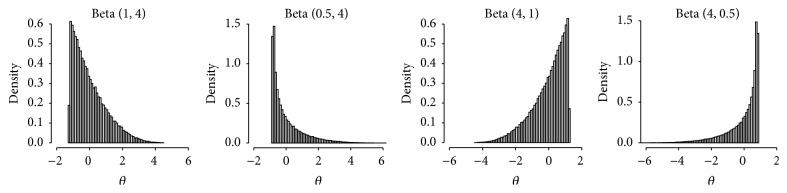
Distribution of the latent trait according to the different parameters of the standardized beta distribution.

As shown in Figure 1, the beta distribution has different shapes depending on the value of these parameters. If *α* was set to 1 (skewed) or 0.5 (highly skewed) and *β* was greater than 1, we obtained a L shaped distribution. Similarly, a J shaped distribution will be obtained when *α* was greater than 1 and *β* was set to 1 (skewed) or 0.5 (highly skewed). The L and J shaped distributions correspond to situations in which participants respond mostly negatively and positively, respectively. In total, we generated 324 (3 × 2 × 3 × 2 × 9) simulation scenarios; each simulated scenario corresponding to a combination of parameters was replicated 1000 times.

## 3. Results

Table 2 shows the power of OLR model under different combinations of sample size ratio, number of response categories, distributions of ability, and magnitudes of DIF when total sample size was 600. Our findings show that the power of OLR model improved as the number of response categories increased. For instance, for the moderate magnitude of DIF (DIF = 0.5), in conditions 1, 2, 3 in which the same ability distribution assumed in both reference and focal groups, increasing the number of response categories from *J* = 3 to *J* = 5 increased the OLR power approximately 10%, 8%, and 6%, when *R* = 1,2, and 3, respectively. However, in conditions 4, 5, 6, 7, 8, and 9, that ability distribution differed in the reference and focal groups; this amount of increment in power was slightly lower and reached approximately 0%, 9%, and 5%, when *R* was equal to 1, 2, and 3, respectively.

The second major finding was that, under various combinations of ability distributions, OLR model had different performances for *J* = 3,4, and 5. The power of OLR model for *J* = 4 was more affected by different distributions of ability than the power of OLR model for *J* = 3 and 5. For example, when *J* = 4, there were decreases of approximately 15%, 23%, and 22% in power for condition 8 as compared with conditions 1, 4, and 6. This amount of reduction was lower for *J* = 3 and 5 which was approximately 9%, 18%, and 20% for *J* = 3 and 12%, 20%, and 19% for *J* = 5. Another example illustrated that when *J* = 4, the power decreased approximately by 11%, 18%, and 18% when the ability distribution of *N*  (0,1) and Beta (0.5,4) for the reference and focal groups was compared to conditions 1, 4, and 6; the comparison of these conditions indicated that the rate of reduction was approximately 9%, 17%, and 16% for *J* = 5 and approximately 2%, 11%, and 14% for *J* = 3.

Regardless of sample size ratio and ability distribution, general comparison of the OLR power among various numbers of response categories indicated that, for moderate magnitude of DIF, power for *J* = 5 was approximately 5.9% and 5.7% higher than *J* = 4 and 3, respectively. In addition, under equal sample size ratio, power was roughly 4% lower for *J* = 4 compared with *J* = 3, while OLR model performed almost similarly for *J* = 3 and *J* = 4 under unequal sample size ratio 3 : 1.

Our findings also indicated that as skewness of ability distribution increased, the power of OLR model decreased. When ability distribution in the reference and focal groups was the same and moderately skewed (condition 2), power is the same or slightly different from the condition in which ability distribution was normal in both groups. In contrast, highly skewed distribution of ability for both reference and focal groups (i.e., conditions 3 and 9) led to inadequate levels of power (less than 0.61), irrespective of sample size ratio and number of response categories. Substantial reduction of approximately 37.7% and 57.7% in power occurred when ability distribution of conditions 3 and 9 was compared to condition 1.

Regarding the effect of unequal sample size ratios, the power of OLR model decreased as the ratio of the inequality increased in all combinations of different numbers of response categories and distributions of ability. Compared with *R* = 1, there were decreases of approximately 7.1% and 17.4% in power for *R* = 2 and 3, respectively. Moreover, when the sample size ratio changed from 2 to 3, power reduced about 11.1%.

When the magnitude of DIF is large (i.e., DIF = 1), power was always close to 1, irrespective of the ratio of sample size, number of response categories, and distribution of ability; even under highly skew distribution of ability in both reference and focal groups, power was greater than or equal to 0.89.

Table 3 presents empirical type I error rate of OLR model at the nominal significance level of 0.05 under various combinations of sample size ratios, distribution of ability, number of response categories, and magnitude of DIF when total sample size was 600. For moderate magnitude of DIF (DIF = 0.5), type I error rate was below or close to the nominal level in all conditions. However, when DIF = 1, type I error rate exceeded the nominal level for some distributions of ability including conditions 5, 7, 8, and 9 which ranged from 0.06 to 0.10.

Table 4 displays the power of OLR model under various combinations of sample size ratio, numbers of response categories, different distributions of ability, and magnitudes of DIF, when total sample size was 1000. When DIF = 0.5, power exceeded 0.79 criterion in all conditions except for highly skewed distribution of ability, namely, conditions 3 and 9. In these cases, power ranged from 0.61 to 0.77 for condition 3 and from 0.39 to 0.61 for condition 9. We also found that unequal sample size ratio led to power reduction irrespective of the distribution of ability and number of response categories; compared with *R* = 1 power decreased approximately by 4% and 10.5% for *R* = 2 and 3, respectively. When the magnitude of DIF is large (DIF = 1), power was 1 or close to 1 in all cases.

Table 5 indicates type I error rate of OLR at the nominal significance level of 0.05 under different conditions when total sample size was 1000. When the magnitude of DIF is moderate, type I error rate was close to 0.05 in all conditions except for the conditions 7, 8 and 9 in which it ranged from 0.05 to 0.08. However, when the magnitude of DIF was large (DIF = 1), type I error rate was higher than the nominal level for almost all conditions and even exceeded 0.1 for conditions 5, 7, 8, and 9.

It should be noted that the results discussed here are based on adding 0.5 to the threshold parameters in focal group to produce uniform DIF. For a limited number of scenarios we subtracted 0.5 from threshold parameters to create uniform DIF and we found out that the power of OLR model substantially changed in some conditions. For instance, under equal sample size ratio, for condition 4 subtracting 0.5 led to reduction in power from 0.89, 0.87, and 0.90 to 0.75, 0.76, and 0.79 when *J* = 3,4, and 5, respectively. While in condition 9 the power increased from 0.35, 0.32, and 0.34 to 0.71, 0.68, and 0.66 by subtracting 0.5 instead of adding 0.5 to the focal group. Due to space limitation explaining about the results of all simulation scenarios regarding the effect of subtracting 0.5 from threshold parameters is beyond the scope of this research.

## 4. Discussion

To the best of our knowledge, this is the first study that has evaluated the effect of the number of response categories on the power of OLR model for detecting DIF. Other factors evaluated in this study were the magnitude of DIF, the ability distribution of the reference and focal groups, the sample size, and the sample size ratios. Regardless of the number of response categories, sample size ratio, and ability distribution, this study showed that, for the large sample size (*N* = 1000) or the large magnitude of DIF (DIF = 1), the power of OLR is 1 or close to 1. Moreover, the effect of the number of response categories on the power of OLR for detecting DIF is slightly influenced by the ratio of sample size in the focal and reference groups.

One of the most obvious findings to emerge from this study was that gains in power from increasing the number of response categories could be affected by the difference between the ability distributions of the focal and the reference groups. Accordingly, when ability level was distributed identically (normal or skewed) in the reference and focal groups, increasing the number of response categories from 3 to 5 resulted in an increase of approximately 8% in the power of OLR model for detecting DIF. Moreover, when the number of response options is kept constant, especially when *J* = 4, the power of OLR can be substantially affected by the ability distribution in the focal and reference groups. We found that, in the case of *J* = 4, the power of OLR model can be reduced by approximately 16% when the ability distribution changes from condition 4 to condition 5.

Another important feature which has been considered in this study is to evaluate whether high level of skewness in ability can affect the power of OLR for detecting DIF regardless of the number of response categories, sample size, and sample size ratio. In most simulation studies, item responses were generated using the GRM which generally assumed normality of the latent ability. However, this assumption may not be encountered in practice. For example, in HRQoL studies, violation of normality assumption can frequently occur when we intend to evaluate measurement equivalence of the instrument across the two diverse groups such as healthy people and people with chronic conditions [18, 19]. In the present research, the simulated distributions were partly extreme. In order to provide evidence as to why these distributions are realistic and relevant to study, readers can be referred to a number of applied and methodological articles in the field of HRQoL. For example, Guilleux et al. evaluated the impact of a deviation from the normality assumption on the performance of the IRT methods used in clinical trials to compare highly skewed latent HRQoL scores across two treatment groups [20]. In addition, Hunger et al. examined the use of beta regression models for analyzing longitudinal HRQoL data in clinical trials and epidemiologic studies when HRQoL scores were highly skewed [21]. Moreover, a large number of papers have dealt with the case that distribution of HRQoL scores was heavily skewed regarding patients with chronic conditions such as asthma, cerebral palsy, and cancer [22–25]. Thus, having considered the volume of research referenced above, it seems reasonable to assume that extreme distributions might happen in practice.

However, in previous simulation studies assessing DIF, limited conditions were considered with respect to ability distributions. In two studies, the ability distribution for the reference group was a standard normal, and the focal group had a distribution that was moderately or highly skewed to the left or right [26, 27]. In another study, a moderate negative skew in ability distribution for both the focal and reference groups was evaluated [28]. All of these studies showed that moderate skewness had very little impact on the power of OLR for DIF detection. However, the most surprising aspect of our study was that compared to when ability level was distributed normally in both groups the power of OLR was reduced approximately by 60% when ability distributions in the reference and focal groups were highly skewed to the left and right, respectively. In this case, increasing the sample size from 300 to 500 per group could not compensate for the reduction in power. Even if ability distribution is highly skewed to the left or right in the focal group, when ability level is normally distributed in the reference group, for *R* = 1 and *J* = 5, OLR model can detect moderate DIF (DIF = 0.5) with a power close to 80%.

In this study, no clear differences in the nominal type I error rate were found under different conditions except when ability distributions were highly skewed in both groups, and also the sample size and the effect of DIF were large. Accordingly, if we intend to draw a general conclusion linking the findings of the current simulation study and the previous ones [8, 11, 26–28], it would be that for the moderate effect of DIF, in terms of type I error rate, OLR is robust to change the number of items in the measure and the number of response categories as well as to moderate skewness of ability distribution.

It should be noted that we have just presented simulations with positive changes in threshold parameters, but corresponding simulations were carried out for negative changes, and the results were totally different when ability distribution in one or both groups was skewed. These findings are different from those in a previous research, which reported that when the ability level in the focal and reference groups were normally distributed, adding or subtracting 0.5 to threshold parameters did not change the results principally [11].

Moreover, in the present simulation study, the number of response categories of the items varied from 3 to 5. However, there is no consensus on the number of response options that would optimize the psychometric properties of the scales. Although some authors argue that reliability is maximized with seven response categories [29, 30], others prefer five-point scale [31, 32]. In practice, the number of response alternatives most frequently used in Likert-type scales is five or even less than five, especially in the field of health and social sciences. For example, KINDL, PedsQL*™* 4.0, and KIDSCREEN are the most frequently used questionnaires in pediatric HRQoL studies with five response options [9, 12, 13]. On the other hand, GHQ-12 and DASS are two measures among a pool of psychological instruments which include four response categories [33, 34]. Moreover, physical functioning subscale in the SF-36 instrument uses 3 response options [35]. To explain why the number of response categories was set to 3, 4, and 5, we also simulated items with 6 and 7 response categories, not reported here in Results. The findings revealed that increasing the number of response categories from 5 to 6 or 7 resulted in an increase of less than 2% in the power of the OLR for DIF detection. With all things considered, it has been preferred to keep the number of response categories limited to 3, 4, and 5.

Similar to any Monte Carlo simulation study, this research had some limitations. One limitation was that just one item with uniform DIF was simulated to avoid the contamination of multiple DIF items in a test. Another limitation was that, we just simulated a hypothetical instrument with 10 items. The result would be different if we simulated more than one item with DIF and increased the number of items in a test.

## 5. Conclusion

The clearest conclusion from this research is that the minimum number of response categories for DIF analysis using OLR should be at least five. However, the impact of the number of response categories in detecting DIF was lower than might be expected. This research provides a guideline to applied researchers in choosing the number of response categories for rating scales in DIF analysis. Moreover, this study revealed that high skewness of ability distributions substantially reduced the power of OLR model to detect uniform DIF, and increasing the sample size could not compensate for the reduction in power. This finding is important because in HRQoL studies it is unrealistic to assume that the ability level is normally distributed across healthy people and people with chronic conditions. In future research, it would be useful to evaluate whether the effect of the number of response categories on OLR power can be influenced by increasing the number of items with DIF. Since increasing the number of items with DIF may contaminate the scale score [8, 36], future research in purification methods is strongly recommended.

## Figures and Tables

**Table 1:** The nine different conditions for latent trait distribution in the reference and focal groups.

Condition	Ability distribution	
	Reference group	Focal group
1	*N* (0, 1)	*N* (0, 1)
2	Beta (1, 4)	Beta (1, 4)
3	Beta (0.5, 4)	Beta (0.5, 4)
4	*N* (0, 1)	Beta (4, 1)
5	*N* (0, 1)	Beta (1, 4)
6	*N* (0, 1)	Beta (4, 0.5)
7	*N* (0, 1)	Beta (0.5, 4)
8	Beta (4, 1)	Beta (1, 4)
9	Beta (4, 0.5)	Beta (0.5, 4)

**Table 2:** The power of OLR model under different combinations when *N* = 600.

Conditions	Ratio	DIF = 0.5			DIF = 1		
		*J* = 3	*J* = 4	*J* = 5	*J* = 3	*J* = 4	*J* = 5
1	*R*1	0.77	0.81	0.84	1.00	1.00	1.00
	*R*2	0.74	0.77	0.79	1.00	1.00	1.00
	*R*3	0.66	0.66	0.69	1.00	1.00	1.00

2	*R*1	0.78	0.79	0.83	1.00	1.00	1.00
	*R*2	0.76	0.76	0.79	1.00	1.00	1.00
	*R*3	0.66	0.69	0.69	1.00	1.00	1.00

3	*R*1	0.53	0.50	0.60	1.00	1.00	1.00
	*R*2	0.43	0.48	0.49	0.98	0.98	0.99
	*R*3	0.38	0.39	0.41	0.96	0.95	0.97

4	*R*1	0.89	0.87	0.90	1.00	1.00	1.00
	*R*2	0.79	0.83	0.86	1.00	1.00	1.00
	*R*3	0.73	0.75	0.79	1.00	1.00	1.00

5	*R*1	0.79	0.73	0.78	1.00	1.00	1.00
	*R*2	0.70	0.71	0.76	1.00	1.00	1.00
	*R*3	0.63	0.63	0.66	1.00	1.00	1.00

6	*R*1	0.87	0.87	0.88	1.00	1.00	1.00
	*R*2	0.85	0.81	0.85	1.00	1.00	1.00
	*R*3	0.76	0.75	0.79	1.00	1.00	1.00

7	*R*1	0.76	0.71	0.78	1.00	1.00	1.00
	*R*2	0.70	0.68	0.70	1.00	1.00	1.00
	*R*3	0.67	0.61	0.63	1.00	1.00	1.00

8	*R*1	0.78	0.71	0.77	1.00	1.00	1.00
	*R*2	0.64	0.66	0.70	1.00	1.00	1.00
	*R*3	0.57	0.53	0.57	1.00	1.00	1.00

9	*R*1	0.35	0.32	0.34	0.96	0.96	0.98
	*R*2	0.28	0.31	0.36	0.95	0.96	0.96
	*R*3	0.27	0.29	0.32	0.89	0.89	0.94

*J*: number of response categories.

*R*1: *n*_*f*_ = 300 and *n*_*r*_ = 300, *R*2: *n*_*f*_ = 200 and *n*_*r*_ = 400, and *R*3: *n*_*f*_ = 150 and *n*_*r*_ = 450.

The conditions are described in Table 1.

**Table 3:** The type I error of OLR model under different combinations when *N* = 600.

Conditions	Ratio	DIF = 0.5			DIF = 1		
		*J* = 3	*J* = 4	*J* = 5	*J* = 3	*J* = 4	*J* = 5
1	*R*1	0.03	0.03	0.03	0.05	0.07	0.07
	*R*2	0.03	0.03	0.03	0.05	0.05	0.06
	*R*3	0.03	0.03	0.03	0.04	0.05	0.05

2	*R*1	0.03	0.03	0.03	0.05	0.06	0.06
	*R*2	0.03	0.04	0.04	0.05	0.05	0.07
	*R*3	0.03	0.04	0.04	0.05	0.04	0.05

3	*R*1	0.03	0.03	0.03	0.04	0.04	0.05
	*R*2	0.03	0.03	0.03	0.04	0.04	0.05
	*R*3	0.03	0.03	0.03	0.04	0.04	0.04

4	*R*1	0.03	0.03	0.03	0.05	0.05	0.05
	*R*2	0.03	0.03	0.03	0.04	0.05	0.05
	*R*3	0.03	0.03	0.03	0.04	0.04	0.04

5	*R*1	0.04	0.04	0.04	0.06	0.07	0.08
	*R*2	0.04	0.04	0.04	0.06	0.07	0.07
	*R*3	0.04	0.04	0.04	0.06	0.06	0.07

6	*R*1	0.03	0.03	0.03	0.07	0.05	0.04
	*R*2	0.03	0.03	0.03	0.04	0.05	0.04
	*R*3	0.03	0.03	0.03	0.04	0.04	0.04

7	*R*1	0.04	0.05	0.05	0.07	0.08	0.09
	*R*2	0.04	0.04	0.05	0.07	0.08	0.08
	*R*3	0.04	0.04	0.04	0.06	0.07	0.07

8	*R*1	0.04	0.05	0.05	0.08	0.09	0.10
	*R*2	0.04	0.06	0.06	0.08	0.09	0.10
	*R*3	0.04	0.05	0.05	0.07	0.08	0.09

9	*R*1	0.06	0.06	0.06	0.07	0.08	0.10
	*R*2	0.05	0.05	0.06	0.06	0.08	0.09
	*R*3	0.04	0.05	0.05	0.06	0.06	0.08

*J*: number of response categories.

*R*1: *n*_*f*_ = 300 and *n*_*r*_ = 300, *R*2: *n*_*f*_ = 200 and *n*_*r*_ = 400, and *R*3: *n*_*f*_ = 150 and *n*_*r*_ = 450.

The conditions are described in Table 1.

**Table 4:** The power of OLR model under different combinations when *N* = 1000.

Conditions	Ratio	DIF = 0.5			DIF = 1		
		*J* = 3	*J* = 4	*J* = 5	*J* = 3	*J* = 4	*J* = 5
1	*R*1	0.97	0.97	0.99	1.00	1.00	1.00
	*R*2	0.94	0.95	0.96	1.00	1.00	1.00
	*R*3	0.88	0.89	0.93	1.00	1.00	1.00

2	*R*1	0.95	0.96	0.98	1.00	1.00	1.00
	*R*2	0.93	0.93	0.94	1.00	1.00	1.00
	*R*3	0.89	0.89	0.90	1.00	1.00	1.00

3	*R*1	0.73	0.73	0.77	0.99	1.00	1.00
	*R*2	0.72	0.71	0.76	1.00	1.00	1.00
	*R*3	0.62	0.61	0.66	1.00	1.00	1.00

4	*R*1	0.98	0.98	0.99	1.00	1.00	1.00
	*R*2	0.97	0.98	0.97	1.00	1.00	1.00
	*R*3	0.94	0.95	0.95	1.00	1.00	1.00

5	*R*1	0.95	0.96	0.97	1.00	1.00	1.00
	*R*2	0.92	0.92	0.94	1.00	1.00	1.00
	*R*3	0.84	0.87	0.89	1.00	1.00	1.00

6	*R*1	0.98	0.98	0.98	1.00	1.00	1.00
	*R*2	0.97	0.98	0.98	1.00	1.00	1.00
	*R*3	0.94	0.96	0.96	1.00	1.00	1.00

7	*R*1	0.94	0.94	0.95	1.00	1.00	1.00
	*R*2	0.92	0.88	0.92	1.00	1.00	1.00
	*R*3	0.85	0.85	0.88	1.00	1.00	1.00

8	*R*1	0.91	0.91	0.94	1.00	1.00	1.00
	*R*2	0.86	0.88	0.92	1.00	1.00	1.00
	*R*3	0.79	0.81	0.85	1.00	1.00	1.00

9	*R*1	0.55	0.54	0.61	0.99	1.00	1.00
	*R*2	0.45	0.45	0.53	0.99	0.99	1.00
	*R*3	0.39	0.40	0.44	0.98	0.98	0.99

*J*: number of response categories.

*R*1: *n*_*f*_ = 500 and *n*_*r*_ = 500, *R*2: *n*_*f*_ = 333 and *n*_*r*_ = 667, *R*3: *n*_*f*_ = 250 and *n*_*r*_ = 750.

The conditions are described in Table 1.

**Table 5:** The type I error of OLR model under different combinations when *N* = 1000.

Conditions	Ratio	DIF = 0.5			DIF = 1		
		*J* = 3	*J* = 4	*J* = 5	*J* = 3	*J* = 4	*J* = 5
1	*R*1	0.03	0.04	0.04	0.07	0.08	0.09
	*R*2	0.04	0.04	0.04	0.07	0.08	0.08
	*R*3	0.03	0.04	0.04	0.05	0.07	0.07

2	*R*1	0.04	0.04	0.04	0.08	0.08	0.09
	*R*2	0.04	0.04	0.04	0.06	0.07	0.08
	*R*3	0.04	0.04	0.04	0.06	0.07	0.08

3	*R*1	0.04	0.04	0.04	0.07	0.06	0.07
	*R*2	0.03	0.03	0.03	0.04	0.05	0.06
	*R*3	0.03	0.03	0.03	0.04	0.05	0.06

4	*R*1	0.03	0.03	0.03	0.05	0.06	0.06
	*R*2	0.03	0.02	0.02	0.05	0.06	0.06
	*R*3	0.03	0.03	0.03	0.05	0.06	0.06

5	*R*1	0.05	0.05	0.06	0.09	0.11	0.12
	*R*2	0.04	0.05	0.05	0.08	0.09	0.10
	*R*3	0.04	0.05	0.05	0.07	0.08	0.09

6	*R*1	0.03	0.03	0.03	0.05	0.06	0.06
	*R*2	0.03	0.03	0.03	0.05	0.06	0.05
	*R*3	0.03	0.03	0.03	0.05	0.06	0.05

7	*R*1	0.05	0.07	0.07	0.10	0.13	0.14
	*R*2	0.05	0.06	0.07	0.10	0.12	0.13
	*R*3	0.05	0.06	0.06	0.08	0.10	0.10

8	*R*1	0.07	0.08	0.08	0.12	0.14	0.16
	*R*2	0.06	0.07	0.08	0.11	0.13	0.14
	*R*3	0.05	0.06	0.06	0.09	0.11	0.12

9	*R*1	0.07	0.07	0.08	0.10	0.11	0.14
	*R*2	0.07	0.07	0.07	0.09	0.11	0.13
	*R*3	0.06	0.06	0.07	0.09	0.10	0.11

*J*: number of response categories.

*R*1: *n*_*f*_ = 500 and *n*_*r*_ = 500, *R*2: *n*_*f*_ = 333 and *n*_*r*_ = 667, and *R*3: *n*_*f*_ = 250 and *n*_*r*_ = 750.

The conditions are described in Table 1.
